# The frequency and severity of epistaxis in children with sickle cell anaemia in eastern Uganda: a case-control study

**DOI:** 10.1186/s12878-017-0085-9

**Published:** 2017-09-07

**Authors:** Amina Nardo-Marino, Thomas N. Williams, Peter Olupot-Olupot

**Affiliations:** 1 Department of Haematology, Herlev and Gentofte Hospital, Copenhagen University Hospital, Herlev Ringvej 75, opgang 1, etage 21, 2730 Herlev, Denmark; 2 London School of Hygiene and Tropical Medicine, London, UK; 3 KEMRI/Wellcome Trust Research Programme, Kilifi, Kenya; 4 Department of Medicine, Imperial College, London, UK; 5 Faculty of Health Sciences, Busitema University, Mbale Campus, Mbale, Uganda; 6 Mbale Clinical Research Institute, Mbale, Uganda

**Keywords:** Sickle cell disease, Sickle cell anaemia, Haemoglobinopathies, Epistaxis, Sub-Saharan Africa

## Abstract

**Background:**

There are a paucity of data on epistaxis as it pertains to sickle cell anaemia. Some case studies suggest epistaxis to be a significant complication in patients with sickle cell anaemia in sub-Saharan Africa; however, no robust studies have sought to establish the epidemiology or pathophysiology of this phenomenon.

**Methods:**

We conducted a case-control study with the aim of investigating the importance of epistaxis among children presenting with sickle cell anaemia at the Mbale Regional Referral Hospital in eastern Uganda. Cases were children aged 2–15 years with an existing diagnosis of laboratory confirmed sickle cell anaemia, while controls were children without sickle cell anaemia who were frequency matched to cases on the basis of age group and gender. The frequency and severity of epistaxis was assessed using a structured questionnaire developed specifically for this study. Odds ratios controlled for age group and gender were calculated using unconditional logistic regression.

**Results:**

A total of 150 children were included, 73 children with sickle cell anaemia and 77 children without sickle cell anaemia. The overall prevalence of epistaxis among children with sickle cell anaemia and children without sickle cell anaemia was 32.9 and 23.4% respectively. The case-control odds ratios for epistaxis, recurrent epistaxis and severe epistaxis were, 1.6 (95%CI 0.8–3.4; *p* = 0.2), 7.4 (1.6–34.5; 0.01), and 8.3 (1.0–69.8; 0.05) respectively.

**Conclusions:**

Our results suggest that in eastern Uganda, children with sickle cell anaemia experience epistaxis more frequently and with greater severity than children without sickle cell anaemia. Further studies are indicated to confirm this conclusion and investigate aetiology.

## Background

Sickle cell anaemia (SCA) is a genetic condition of growing public health importance in sub-Saharan Africa (SSA), where an estimated 250,000 children are born with the disease every year [1]. SCA is associated with severe acute and chronic illness and contributes significantly to childhood morbidity and mortality in the region [2]. While epistaxis is not a well-recognized complication of SCA in developed country settings, some authors have suggested that it might be a significant problem in children living with SCA in SSA. Although frequent and severe episodes of epistaxis could potentially be associated with significant complications in this group of patients, no previous studies have examined epistaxis as a primary outcome variable among patients with SCA.

Epistaxis is defined as acute haemorrhage from the nostril, nasal cavity or nasopharynx [3]. In general, data regarding the prevalence of epistaxis among children living in SSA are inadequate, as compared to similar studies in European and North American populations. Nevertheless, it is widely recognised that epistaxis is common. For example, Petruson has estimated that 30, 56 and 64% of children among the age groups < 5 years, 6–10 years and 11–15 years respectively, have experienced at least one episode [4]. The severity of epistaxis can range from a single short-lived episode to, less commonly, severe and life-threatening haemorrhage requiring urgent medical intervention [5]. In the majority of cases of childhood epistaxis, the bleeding originates from the venous plexus of Kiesselbach located on the anterior nasal septum. The bleeding is usually self-limiting and is most often caused by digital trauma or crusting [6, 7]. It has previously been hypothesised that recurrent epistaxis in patients with SCA might be associated with hypersplenism and thrombocytopaenia [8] or could possibly be caused by a thromboinfarctive process in the nasal mucous membrane over Kiesselbach’s area [9]. These theories, however, have not been confirmed.

Data on epistaxis as it pertains to SCA, as well as other types of sickle cell disease (SCD), are limited and results of previous studies require extrapolation. Past estimates of the prevalence of epistaxis in patients with SCA and SCD range from 5.3 to 35.1% [8–12]. These estimates originate from study populations differing enormously in geography, phenotype, and age. There is a common lack of comparative data from patients without SCA and, to the best of our knowledge, only one previous study has described a gradient of severity amongst patients with SCA experiencing epistaxis [13]. Therefore, it remains difficult to correlate existing data with severity and clinical outcomes. In the current study, we aimed to determine whether children with SCA in SSA experience more frequent or more severe episodes of epistaxis than children without SCA, potentially laying the groundwork for larger studies examining this question in further detail.

## Methods

### Study location

The study was conducted at the Mbale Regional Referral Hospital (MRRH), located in the mid-eastern region of Uganda. MRRH is a public referral hospital that admits approximately 17,000 paediatric patients every year. With the prevalence of SCA at birth being as high as 1.2% within the region [14], children with SCA constitute a large part of paediatric in- and outpatient practise within the hospital.

### Study design and participants

The study was carried out during July and August 2016. We conducted a case-control study in which cases were children aged 2–15 years with an existing diagnosis of laboratory confirmed SCA, based on haemoglobin electrophoresis. Case children with SCA were recruited from the weekly SCA outpatient clinic or from the paediatric wards at MRRH. Control children without SCA were recruited from the general paediatric outpatient clinic or from among patients or visitors at the paediatric wards at MRRH. Controls were frequency matched to cases on the basis of age group and gender. Both children with SCA and children without SCA were selected randomly from within the clinic and ward facilities. As this was a pilot study the sample size chosen was pragmatic. We aimed to include a minimum of 150 participants.

The primary outcomes measured were the prevalence of ≥1 episode of epistaxis and the frequency and severity of epistaxis among children with SCA and children without SCA. Any children with an existing condition associated with epistaxis, including bleeding disorders, bone marrow or liver dysfunction, haematological malignancies, severe acute malnutrition, tuberculosis, hepatitis, and HIV positivity, were excluded as participants, as were children who were already enrolled in other studies. Furthermore, children receiving treatment with bone marrow modifying drugs, such as hydroxyurea, were not included in the study.

### Questionnaire

We developed a structured questionnaire on the frequency and severity of epistaxis specifically for this study. Prior to beginning data collection, a test survey with 15 children and their parents or guardians was conducted with the purpose of testing our questionnaire for coherency and to ensure quality control. This test survey resulted in minor changes to the questionnaire, primarily affecting the order and wording of questions. To ensure consistency, all interviews were carried out by the first author (ANM). If neither child nor parent or guardian was able to speak English, a member of the local research team helped with translation. During the interview process, it was ensured that both the child and their parent or guardian were involved.

### Definitions

Epistaxis was defined as “recurrent” if the child or parent or guardian reported ≥ 5 episodes of epistaxis during the child’s life. An episode of epistaxis was defined as “heavy” if the bleeding was described as profound and first aid measures, such as compression or nasal packing, had been necessary in order to terminate the bleeding. Epistaxis was defined as “severe” if the child or parent or guardian reported, heavy bleeding, if a an episode of epistaxis lasted > 30 min if compression or nasal packing was not applied, or if the child had received a blood transfusion as a direct consequence of an episode of epistaxis.

### Ethical considerations

The Mbale Regional Referral Hospital Research and Ethics Committee and the London School of Hygiene and Tropical Medicine Research Ethics Committee granted ethical approval for this study. Written, informed consent from a parent or guardian over 18 years and verbal assent from all children old enough to understand plus written assent from all children over 8 years was obtained before recruitment. Study participants did not receive any compensation for their participation in the study. As part of the study, all children and their parents or guardians were instructed in simple first aid measures to terminate nose bleeding. Moreover, parents or guardians of children with recurrent or severe epistaxis were instructed on how to perform basic nasal packing at home and encouraged to do so accordingly.

### Statistical analysis

Data were analysed in Stata V14.1 [StataCorp, Timberlake, USA]. Odds ratios (ORs) controlled for age group and gender were calculated using unconditional logistic regression. An unmatched statistical approach was chosen over a matched analysis as frequency matching was performed, as opposed to a pair matched design [15].

## Results

**Fig. 1:**
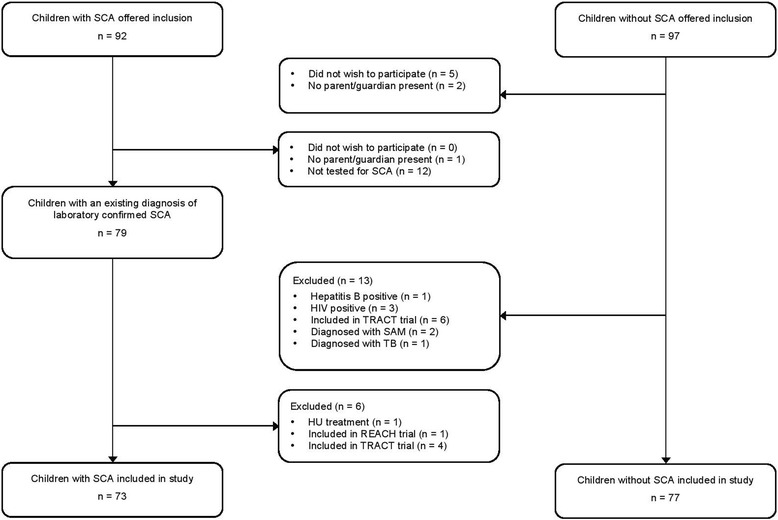
Flowchart of participants included in and excluded from the study. HIV: Human Immunodeficiency Virus, HU: Hydroxyurea, REACH: Realizing Effectiveness Across Continents with Hydroxyurea, SAM: Severe Acute Malnutrition, SCA: Sickle Cell Anaemia, TB: Tuberculosis, TRACT: Transfusion and Treatment of severe Anaemia in African Children

A total of 150 children were included in the study, 73 children with SCA and 77 children without SCA. Figure 1 illustrates the flow of children included in and excluded from the study*.* Thirty-four (46.6%) of the children with SCA and 38 (49.4%) of the children without SCA were female. Ages ranged from 2 to 15 years with a mean age of 7.4 years in both groups. The overall prevalence of epistaxis (≥ 1 episode) was 32.9% among children with SCA and 23.4% among children without SCA. The prevalence of epistaxis among children with SCA and children without SCA, stratified by age group and gender, is summarised in Table 1.

**Table 1:** Prevalence of epistaxis (≥ 1 episode) in children with and without SCA, stratified by age group and gender

	Children with SCA reporting epistaxis/total (%)	Children without SCA reporting epistaxis/total (%)
Total	24/73 (32.9)	18/77 (23.4)
Gender		
Female	13/34 (38.2)	8/38 (21.1)
Male	11/39 (28.2)	10/39 (25.6)
Age (years)		
2–5	6/25 (24)	3/27 (11.1)
6–9	9/26 (34.6)	5/27 (18.5)
10–15	9/22 (40.9)	10/23 (43.5)

*SCA* sickle cell anaemia

Of the 24 children with SCA reporting epistaxis, 12 (50%) had experienced recurrent epistaxis (≥ 5 episodes) and 7 (29.2%) reported severe epistaxis. In comparison, only 2 (11.1%) of the 18 control children without SCA who reported epistaxis had experienced recurrent epistaxis and only 1 (5.6%) reported severe epistaxis. When controlled for age group and gender, the ORs for epistaxis, recurrent epistaxis and severe epistaxis in children with SCA compared to children without SCA were found to be, 1.6 (95%CI 0.8–3.4; *p* = 0.2), 7.4 (1.6–34.5; 0.01), and 8.3 (1.0–69.8; 0.05) respectively. The characteristics of epistaxis in children with and without SCA are summarised in Table 2.

**Table 2:** Characteristics of epistaxis in children with and without SCA

	Children with SCA No. (%)	Children without SCA No. (%)	OR^a^	95% CI	*p*-value
Epistaxis (≥ 1 episode)	24 (32.9)	18 (23.4)	1.6	0.8–3.4	0.2
Recurrent epistaxis (≥ 5 episodes)	12 (16.4)	2 (2.6)	7.4	1.6–34.5	0.01
Severe epistaxis	7 (9.6)	1 (1.3)	8.3	1.0–69.8	0.05
Duration > 30 min	4 (5.5)	1 (1.3)			
Heavy bleeding	4 (5.5)	0			
Blood transfusion as a consequence of epistaxis	3 (4.1)	0			

*SCA* sickle cell anaemia. ^a^Controlled for age group and gender

Of the children with epistaxis, only 3 reported episodes that were secondary to trauma: 1 child with SCA and 2 children without SCA. Each had experienced a single episode of epistaxis, all of mild intensity lasting less than 5 min. Furthermore, 16 of the 24 children with SCA and epistaxis (66.7%) reported bleeds to be associated with episodes of febrile illnesses and/or painful crises.

## Discussion

We conducted a case-control study addressing the frequency and severity of epistaxis among children with SCA, compared to age and gender matched control children without SCA, in order to investigate the importance of epistaxis among children with SCA in eastern Uganda.

When comparing rates of epistaxis among children with and without SCA, we found that SCA was associated with a 1.6-fold increase in the odds of experiencing epistaxis (≥ 1 episode). Furthermore, SCA was associated with a 7.4-fold increase in the odds of experiencing recurrent epistaxis (≥ 5 episodes) and a 8.3-fold increase in the odds of experiencing severe epistaxis. Despite the fact that only the results for recurrent epistaxis were statistically significant, these findings suggest that SCA may well be a risk factor for experiencing both more frequent episodes of epistaxis as well as severe epistaxis, with the lack of significance possibly resulting from limited power. Frequent and severe episodes of epistaxis could be associated with severe anaemia in children with SCA [16] and the implementation of patient and parent education regarding appropriate treatment of epistaxis could potentially alter clinical outcomes for some children. In this study, epistaxis was found to be associated with episodes of febrile illness and painful crises in 68% of children with SCA. Nevertheless, the aetiology remains unclear.

Among the case children with SCA, the overall prevalence of epistaxis (≥ 1 episode) was 32.9%, with the prevalence of epistaxis in children aged 2–5, 6–9 and 10–15 years being 24, 34.6, and 40.9% respectively. Having assessed the prevalence of epistaxis among 591 paediatric SCA patients in the Democratic Republic of the Congo, Tshilolo and colleagues presented data estimated in a population similar to the one included in this study [11]. They found that 6.5, 5.8 and 17.4% of inpatient children with SCA aged 3–5, 6–12, and > 13 years respectively, reported epistaxis, as well as 26% of outpatient children with SCA. Similarly, Konotey-Ahulu reported that 106 of 1345 patients (7.9%) enrolled in a SCD clinic in Accra, Ghana, had experienced epistaxis during a 12-year period of observation [9]. These estimates are low compared to the prevalence of epistaxis found in this study. Unfortunately, the lack of a clear definition of epistaxis as an outcome measure in both studies makes it difficult to compare results.

### Study limitations

None of the children without SCA who were included in the study had presented with clinical symptoms compatible with the disease. However, we cannot discount entirely the possibility of misclassification, as the diagnosis was not excluded by formal testing.

Although all the interviews were conducted by a single investigator, many study participants did not speak English and help from a translator was often required. Thus, the conformity of the interviews was compromised and information bias possibly introduced to the study. It was assumed that most children diagnosed with SCA were frequently in contact with the paediatric department at the MRRH due to severe clinical manifestations of their disease. Therefore, selecting children with SCA entirely from the MRRH should have resulted in a group of cases representative of the general SCA population in the area. However, comparability between children without SCA included in the study and the general paediatric population may have been affected by solely including children from the hospital. Thus, it is possible that the prevalence of epistaxis among children without SCA is in fact an overestimation.

## Conclusions

The results from this study suggest that children with SCA in eastern Uganda experience more frequent and severe episodes of epistaxis than children without SCA. Furthermore, the results suggest that epistaxis in children with SCA is somehow related to episodes of febrile illnesses and painful crises. Recurrent and severe epistaxis could potentially result in severe anaemia in patients with SCA and, in order to implement necessary patient care, further studies are indicated to confirm this conclusion and investigate aetiology.

## Abbreviations

MRRH: Mbale Regional Referral Hospital
OR: Odds Ratio
SCA: Sickle Cell Anaemia
SCD: Sickle Cell Disease
SSA: Sub-Saharan Africa
